# Increased Prevalence of Colorectal Polyp in Acromegaly Patients: A Case-Control Study

**DOI:** 10.1155/2014/152049

**Published:** 2014-12-29

**Authors:** Ali Riza Koksal, Meltem Ergun, Salih Boga, Huseyin Alkim, Mehmet Bayram, Yuksel Altuntas, Banu Ozguven Yilmaz, Canan Alkim

**Affiliations:** ^1^Gastroenterology Clinic, Sisli Etfal Education and Research Hospital, 34360 Istanbul, Turkey; ^2^Endocrinology Clinic, Sisli Etfal Education and Research Hospital, 34360 Istanbul, Turkey; ^3^Pathology Clinic, Sisli Etfal Education and Research Hospital, 34360 Istanbul, Turkey

## Abstract

An increase in the prevalence of colorectal polyps and cancer is reported in patients with acromegaly. This trial is designed to determine whether there is an increase in the prevalence of colorectal polyps/cancer in Turkish acromegaly patients. Sixty-six patients, who were under follow-up with the diagnosis of acromegaly and underwent total colonoscopic examination, were enrolled in the study. Sixty-five age- and gender-matched patients with nonspecific complaints were selected as control. The mean age of acromegalic patients was 51.5 ± 12.8 years of whom 27 (40.9%) were females. In 20 (30.3%) of the patients with acromegaly a total of 65 colorectal polyps were detected. Forty-seven (72.3%) of the polyps were detected at the rectosigmoid region. In 8 (12.3%) of the 65 control patients a total of 17 polyps were found. There was a statistically significant difference between the groups (*P* = 0.018). At the logistic regression analysis we found that the risk for colon polyps increased 3.2-fold in the presence of acromegaly, irrespective of age and gender (OR: 3.191, 95% CI: 1.25–8.13). In conclusion, patients who were followed up with the diagnosis of acromegaly should be taken to the colonoscopic surveillance program and all polyps detected should be excised in order to protect them from colorectal cancer.

## 1. Introduction

Acromegaly is a disorder which usually results from a pituitary adenoma and is manifested with the increased circulating levels of growth hormone (GH) and insulin-like factor type 1 (IGF-1) and is characterized by overdevelopment of the distal bones, soft tissue, and the internal organs. The life expectancy is usually shorter than the normal population due to cardiovascular, pulmonary, and cerebrovascular causes [1].

Recent studies report a higher prevalence of colorectal cancer and polyp in acromegaly patients compared to the normal population [2–10]. Colorectal cancer, one of the most common cancers observed in the overall population, is known to occur highly on the basis of adenomatous polyps. Detection and removal of polyps via colonoscopic screening were proven to reduce the mortality associated with colorectal cancer [11, 12]. Since colorectal polyps and cancers are considered to develop earlier due to the effects of increased GH and IGF-1 in acromegaly patients, it may be appropriate to perform a colonoscopy screening in acromegaly patients more frequently and earlier relative to the overall population. Therefore, certain guidelines indicate the need for colonoscopic monitoring performed every 3 to 5 years, starting from the age of 40 in acromegaly patients [13, 14]. In our country, there are a few publications on the prevalence of colorectal polyps among patients with acromegaly [15, 16].

In this study, by comparing the results obtained from the patients undergoing colonoscopy due to acromegaly to those from the age- and gender-matched control subjects, we aim to determine whether there is an increase in the prevalence of colorectal polyps/cancer in Turkish acromegaly patients.

## 2. Material and Methods

### 2.1. Study Design

Sixty-six acromegaly patients, who underwent total colonoscopy for the first time between January 2011 and May 2014 at the Gastroenterology Endoscopy Unit of the Şişli Etfal Education and Research Hospital, were enrolled in the study. All of the patients were under the follow-up of our hospital endocrinology clinic. Diagnosis of acromegaly was based on IGF-1 level measurement, GH suppression test with 75 gr oral glucose load, and hypophyseal magnetic resonance imaging. At this retrospective study, demographic, colonoscopic, and histopathological results of the patients were obtained from the hospital computer database. Sixty-five randomly selected age- and gender-matched patients who underwent colonoscopy for the first time during the same period not due to established reasons such as inflammatory bowel disease, colorectal polyp/cancer follow-up but for causes such as irregular bowel movement, abdominal pain, constipation, and distension were included as the control subjects.

### 2.2. Colonoscopic and Histopathological Assessment

Colonoscopy was performed by 4 experienced endoscopists using an Olympus Exera-II 180 CV colonoscope. All the polyps detected during the procedure were resected with a hot biopsy forceps or snare. Data on the number and size of the resected polyps were obtained from the colonoscopy reports. Only patients with a total colonoscopic examination up to the cecum were included to the study. For performing a histopathological assessment, respective paraffin blocks of patients were reached from the pathology archive and the assessment was performed by a single pathologist.

### 2.3. Statistical Analysis

Statistical analyses were conducted using SPSS version 21.0 (Chicago, IL) software. The compliance of the data to the normal distribution was evaluated via visual (histogram) and analytical methods (Kolmogorov-Smirnov). Mann Whitney *U* and Fisher's exact tests were used for comparing the patient and the control groups. The categorical variables of gender and the presence of acromegaly as well as the continuous variables of age were used in the logistic regression analysis. The odds ratio was calculated for all the variables. All the analyses were performed within the 95% confidence range and *P* < 0.05 was considered statistically significant.

## 3. Results

A total of 66 acromegaly patients, including 27 females (40.9%) and 39 males (59.1%), were included to the study. The control group consisted of 29 female (44.9%) and 36 (55.4%) male patients. The mean age in the patient group was 51.5 ± 12.8 years. The median disease age of acromegaly patients was 4 years (range between 2 and 11) (Table 1).

**Figure 1:**
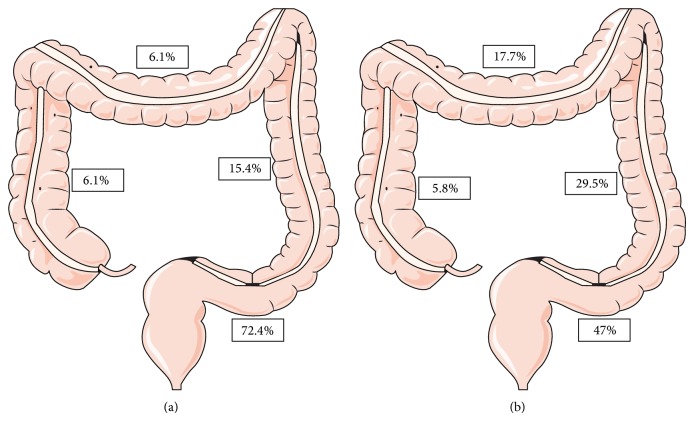
The rates of polyp localization in the acromegaly and control groups: (a) acromegaly group and (b) control group.

In 20 (30.3%) of the patients with acromegaly a total of 65 colorectal polyps were detected. In 8 (12.3%) of the 65 patients in the control group a total of 17 polyps were found. There was a statistically significant difference between the polyp frequencies of the two groups (*P* = 0.018). In 20 patients with acromegaly, 65 colorectal polyps were detected and resected in total. Forty-seven (72.3%) of the polyps were detected in the rectosigmoid region, 10 (15.3%) in the descending colon, 4 (6%) in the transverse colon, and 4 (6%) in the right colon. As for the 17 polyps detected in 8 patients in the control group, 8 (47%) of the polyps were detected in the rectosigmoid region, 5 (29.5%) were in the descending colon, 3 (17.7%) were in the transvers colon, and 1 (5.8%) was in the right colon (Figure 1). There was a statistically significant difference between the two groups with respect to the rate of polyps detected at the rectosigmoid region (*P* = 0.045).

There was a statistically nonsignificant difference between the female (22.2%) and male (35.9%) genders with respect to the presence of polyps in the acromegaly group. But at the control group, while 19.4% of the males were detected to have polyps, only 3.4% of the females had polyps. This difference was close to statistical significance (*P* = 0.051).

In the histopathological evaluation 42 (64.6%) of the 65 polyps detected in the acromegaly group were reported as adenomatous and the remaining 23 (35.4%) polyps were reported as hyperplastic. At the control group, 10 (58.8%) of the 17 polyps were adenomatous and the remaining 7 (42.2%) were hyperplastic. This histopathological distribution was similar between the two groups. While 18 of the 66 patients (27.3%) had adenomatous polyps in the acromegaly group, 6 of the 65 patients (9.2%) were detected to have adenomatous polyps in the control group (*P* = 0.012). While 16 (24.2%) patients in the acromegaly group had hyperplastic polyps, 5 patients in the control group (7.7%) had hyperplastic polyps (*P* = 0.016).

The mean number of polyps was 3.35 ± 1.5 and the mean largest polyp diameter was 5.4 ± 3.4 mm in the acromegaly group. Same values for the control group were 2.1 ± 1 and 6.8 ± 2.4 mm, respectively (*P* > 0.05). Only one high-grade dysplasia was detected in a 15 mm polypectomy material in the acromegaly group. No adenocarcinoma was detected. None of the patients in the control group had high-grade dysplasia. At the logistic regression analysis we found that the risk for colon polyps increased 3.2-fold in the presence of acromegaly, irrespective of age and gender (OR: 3.191, 95% CI: 1.25–8.13) (Table 2).

The prevalence of colonic diverticula was 12.1% and 10.8% in the acromegaly patients and control patients, respectively. There was no statistically significant difference between the two groups.

## 4. Discussion

The prevalence of benign and malignant tumors is known to be increased in acromegaly patients. In a relevant study, the prevalence of benign and malignant tumors was investigated in 101 acromegaly patients. In this group, the prevalence for the thyroid nodules, uterine polyps, prostate adenomas, colonic polyps, thyroid cancer, endometrium cancer, cervical cancer, and colon cancer was 63%, 4%, 2%, 13.1%, 3%, 3%, 3%, and 2%, respectively [17]. In 1982, Klein et al. reported that the prevalence of colonic polyps was increased among acromegaly patients [2]. In this trial, 5 of the 17 patients (29.4%) were detected to have adenomatous polyps. Subsequently, other literature studies investigating the polyp prevalence among the acromegaly patients reported different rates ranging between 22% and 38% [3–10]. In our study, we detected a colonic polyp prevalence of 30.3% in acromegaly patients, in line with the literature rates. This rate was significantly higher than that of the age- and gender-matched control subjects without acromegaly (12.3%). In our study, we found that the presence of acromegaly increased the risk for colonic polyps 3.2-fold, irrespective of age and gender.

In a meta-analysis including 9 studies [7], the prevalence of adenomatous and hyperplastic colonic polyps was 23% and 22.3%, respectively, among acromegaly patients. These rates were statistically higher than those of the control group. In our study, we detected high rates for both types of polyps in acromegaly patients, in line with this data. In their study, Jenkins et al. detected that adenomas located in the left colon were 4.2-fold more frequent in the acromegaly group compared to the control group [6]. In our study, 72.4% of the polyps in the acromegaly group were detected at the rectosigmoid region while this rate was significantly lower in the control group (47%). Even if this result appears to be different, it is obvious that our patients are detected to have an approximately 2-fold higher rate of polyps in the distal sites of the colon, compared to the control group.

In a retrospective Turkish study about the prevalence of colonic polyps in the general population, 894 of the 6250 persons (14.3%) were detected to have a colon polyp and 47% of these polyps were observed to be located in the rectosigmoid region [18]. These results are similar to the results of the control group in our trial (12.3% and 47%, resp.). This suggests that our control group reflects the characteristics of the overall Turkish population. In a Turkish trial by Berker et al., the prevalence of polyps was detected to be 30.2% in the acromegaly patients and 10% in the control group [16]. This data is also similar to our results.

In a trial by Wassenaar et al. [19], the prevalence of colonic diverticula was observed to be statistically higher in the acromegaly patients compared to age- and gender-matched controls (37% versus 19%, *P* = 0.002). In our study, we also observed a higher prevalence of colonic diverticula in the acromegaly group relative to the control group. However, this difference did not reach statistical significance (22.7% versus 10.8%, *P* = 0.067).

In conclusion, the prevalence of colonic adenomatous and hyperplastic polyps is higher in the acromegaly patients compared to the normal population in our country. To ensure effective protection against colon cancer, patients who are under follow-up due to acromegaly should be enrolled in a surveillance program at an early stage and undergo polypectomy for all the polyps detected during colonoscopy.

## Figures and Tables

**Table 1:** Characteristics of the study population.

	Acromegaly group (*n* = 66)	Control group (*n* = 65)	*P* value
Age (mean ± SD)	51.5 ± 12.8	51.4 ± 12.9	0.97
Gender			
Female	27 (40.9%)	29 (44.6%)	0.66
Male	39 (59.1%)	36 (55.4%)	
Polyp			
Yes	20 (30.3%)	8 (12.3%)	0.018
No	46 (69.7%)	57 (87.7%)	
Diverticula			
Yes	15 (22.7%)	7 (10.8%)	0.067
No	58 (87.9%)	58 (89.2%)	
Number of polyps (mean ± SD)	3.2 ± 1.5	2.1 ± 0.9	0.06
The largest polyp diameter mm/(mean ± SD)	5.4 ± 3.4	6.8 ± 2.4	0.64

**Table 2:** The results of the logistic regression analysis for the presence of acromegaly and the polyp risk.

	OR	95% CI		*P* value
		Lower bound	Upper bound	
Age	1.036	0.998	1.076	0.64
Gender (male)	2.436	0.919	6.454	0.73
Presence of acromegaly	3.191	1.252	8.131	0.015
